# Unique fractal evaluation and therapeutic implications of mitochondrial morphology in malignant mesothelioma

**DOI:** 10.1038/srep24578

**Published:** 2016-04-15

**Authors:** Frances E. Lennon, Gianguido C. Cianci, Rajani Kanteti, Jacob J. Riehm, Qudsia Arif, Valeriy A. Poroyko, Eitan Lupovitch, Wickii Vigneswaran, Aliya Husain, Phetcharat Chen, James K. Liao, Martin Sattler, Hedy L. Kindler, Ravi Salgia

**Affiliations:** 1 Department of Medicine, Section of Hematology/Oncology and the Comprehensive Cancer Center, University of Chicago, Chicago, IL, 60637, USA; 2 Department of Cell and Molecular Biology, Feinberg School of Medicine, Northwestern University, Chicago, IL, 60611, USA; 3 Department of Pathology, University of Chicago, Chicago, IL, 60637, USA; 4 Department of Medical Oncology and Therapeutics Research, City of Hope, Duarte, CA 91010, USA; 5 University of Maryland, College Park, MD, 20742, USA; 6 Department of Surgery, University of Chicago, Chicago, IL, 60637, USA; 7 Department of Medicine, Section of Cardiology, University of Chicago, Chicago, IL, 60637, USA; 8 Department of Medicine, Hematology/Oncology, Dana-Farber Cancer Institute, Boston, MA, 02215, USA

## Abstract

Malignant mesothelioma (MM), is an intractable disease with limited therapeutic options and grim survival rates. Altered metabolic and mitochondrial functions are hallmarks of MM and most other cancers. Mitochondria exist as a dynamic network, playing a central role in cellular metabolism. MM cell lines display a spectrum of altered mitochondrial morphologies and function compared to control mesothelial cells. Fractal dimension and lacunarity measurements are a sensitive and objective method to quantify mitochondrial morphology and most importantly are a promising predictor of response to mitochondrial inhibition. Control cells have high fractal dimension and low lacunarity and are relatively insensitive to mitochondrial inhibition. MM cells exhibit a spectrum of sensitivities to mitochondrial inhibitors. Low mitochondrial fractal dimension and high lacunarity correlates with increased sensitivity to the mitochondrial inhibitor metformin. Lacunarity also correlates with sensitivity to Mdivi-1, a mitochondrial fission inhibitor. MM and control cells have similar sensitivities to cisplatin, a chemotherapeutic agent used in the treatment of MM. Neither oxidative phosphorylation nor glycolytic activity, correlated with sensitivity to either metformin or mdivi-1. Our results suggest that mitochondrial inhibition may be an effective and selective therapeutic strategy in mesothelioma, and identifies mitochondrial morphology as a possible predictor of response to targeted mitochondrial inhibition.

Malignant mesothelioma (MM) is an aggressive disease for which overall outcome is quite poor. The median survival for MM being just 12 months^1^. Although the use of asbestos has decreased in recent years, its latency period, which can be up to 40 years, means large numbers of new MM patients are still diagnosed each year^1^,^2^,^3^. In the US approximately 3000 new cases are diagnosed each year, with the majority of them being advanced stage. Three histologies are usually identified in MM: epithelioid, which is the most common, biphasic, and sarcomatoid^4^. There are a number of immunohistochemical markers such as WT-1 and calretinin that differentiate mesothelioma from other tumors such as lung cancer^5^. In certain subsets of MM, one can detect circulating mesothelin and osteopontin in patient serum^6^. Recently, a number of genetic alterations in BAP1 and NF2 have been identified, that may be prognostic and potentially predictive of therapeutic response^3^,^7^,^8^,^9^,^10^. As an example, loss or mutation of merlin (NF2) may be a predictor of effective targeting by anti-focal adhesion kinase (FAK) therapy^11^. The standard of care in mesothelioma remains surgery or combination chemotherapy with pemetrexed and cisplatin^7^. Although new therapies targeting the immune system, PI3kinase and mTOR are emerging more options are needed if improved outcomes and increased survival are to become a reality for MM patients^12^,^13^,^14^.

In MM layering of the pleura leads to the formation of a solid tumor structure^4^. However tumors are not easily quantified by the conventional metrics of length or volume, therefore we examined the fractal properties of the tumor structure. Fractals are mathematical constructs, which exhibit self-similarity over an infinite scale^15^,^16^. Many biological structures are considered to have fractal properties whereby they exhibit self-similarity within a limited scaling window, often 2–3 orders of magnitude^17^. Objects exhibiting exact, quasi, or statistical self-similarity may be considered fractal. Fractal dimension measurements can be used to indicate the complexity and space-filling properties of a shape^18^,^19^,^20^,^21^. Lacunarity is another measurement often used in conjunction with fractal dimension to describe the texture of a shape or fractal^22^,^23^. In this study fractal dimension and lacunarity measurements were leveraged to differentiate between benign and malignant MM tissues and to classify the different mitochondrial morphologies exhibited by mesothelioma cell lines.

Mitochondria form a dynamic network within the cell, which allows them to respond and adapt as the cell progresses through the cell cycle and to withstand cell stresses such as increased energy demand, nutrient deprivation or hypoxia^24^,^25^,^26^. Mitochondrial networks are often classified as predominately elongated, fragmented or reticulated^27^,^28^. These classifications are indicative of the relative rates of fission and fusion occurring within the network and may alter depending of the state of the cell. Mitochondrial dynamics (cycling between mitochondrial fission and fusion) help to maintain mitochondrial integrity and functional ability^29^. At various points within the normal cell cycle the mitochondria may undergo increased rates of fission (G2-M) or fusion (G1-S)^25^. Increases in fission are also observed during the initial stages of apoptosis, while increased fusion may aim to preserve mitochondrial function in response to cell stress such as hypoxia and cytotoxicity^27^,^30^,^31^. In the current study we have investigated mitochondrial morphology in MM cell lines and quantified the various morphologies using fractal dimension and lacunarity. We have examined the functional outputs of the various mitochondrial morphologies by measuring the metabolic activity in these cells. Oxidative phosphorylation and glycolysis were measured via oxygen consumption rate (OCR) and extracellular acidification rate (ECAR) respectively. Mitochondrial stress testing allowed us to calculate the oxidative reserve capacity of these cells^32^. Finally we examined the sensitivity of MM cell lines to conventional chemotherapeutics (cisplatin) and to the mitochondria targeted inhibitors metformin and mdivi-1. Our results indicate that mesothelioma cells show a spectrum of mitochondrial morphologies ranging from elongated, highly reticulated in H2373 and H2596 cells, to a more fragmented and condensed pattern in H28 and H513. These differences were easily and objectively quantified using the fractal dimension and lacunarity measurements. The MM cell lines exhibited a range of metabolic phenotypes, exhibiting increased reliance on glycolytic function (as indicated from the OCR/ECAR ratio) compared to MeT-5A control mesothelial cells, (with the exception of the H2373 and H2461 lines). Mitochondrial morphology showed better correlation with mitochondrial inhibitor sensitivity that metabolic function measured via oxygen consumption rate (OCR) and extracellular acidification rate (ECAR).

## Results

### Morphology of malignant mesothelioma subtypes

**Figure 1:**
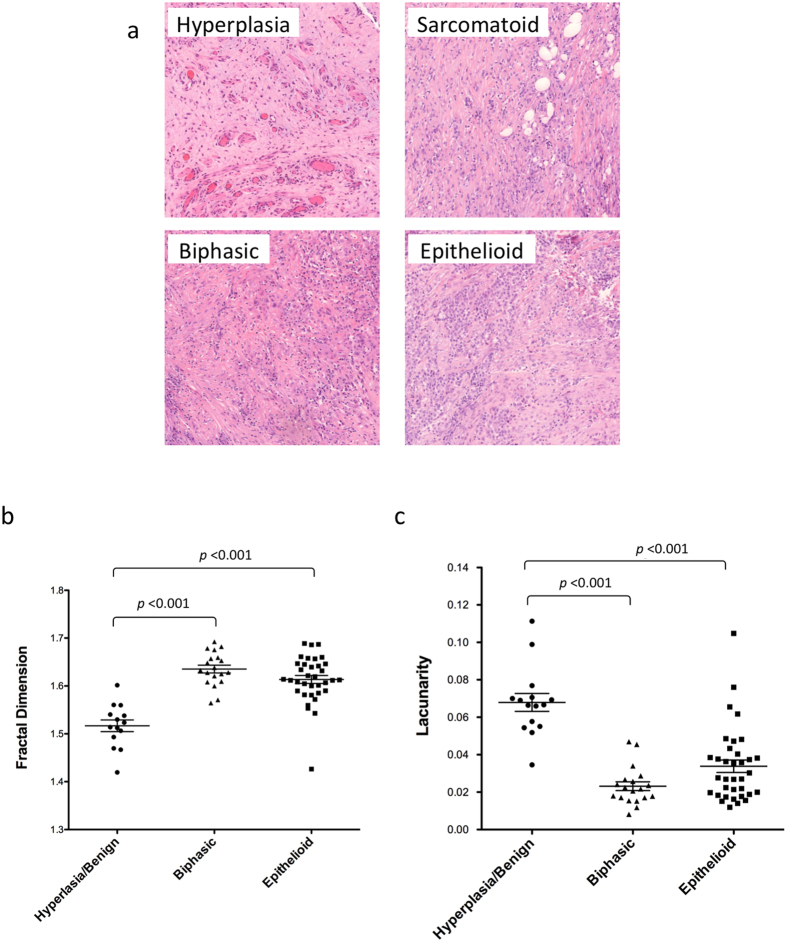
Fractal dimension and lacunarity of mesothelioma subtypes. (**a**) Hematoxylin and eosin-stained sections of hyperplastic mesothelium and various malignant pleural mesothelioma subtypes, the (**b**) fractal dimension and (**c**) lacunarity of hyperplastic/benign, biphasic and epithelioid tumor samples was calculated using the FracLac ImageJ plug-in. Significant differences were detected between hyperplasia/benign control tissues and the biphasic and epithelioid subtypes, *p* ≤ 0.001. No significant different was detected between the biphasic and epithelioid subtypes. Results were analyzed via ANOVA with Tukey post-test. and are shown as the mean and SEM of each subtype.

We have utilized fractal dimension and lacunarity analysis as a novel method to differentiate between hyperplastic and malignant mesothelial tissue^18^,^33^. A tissue microarray (TMA) was constructed containing duplicate samples of hyperplastic, biphasic and epithelioid mesothelium (Fig. 1a). The hematoxylin and eosin (H&E) stained TMA was then scanned using a 3D- Histech Pannoramic SCAN whole slide scanner and images were analyzed (at 15x magnification) using the ImageJ plugin FracLac to calculate the fractal dimension and lacunarity of each sample. Due to some missing or damaged samples the resulting number of tumors analyzed was 36 epithlioid, 20 biphasic and 12 hyperplastic/benign controls. As shown in Fig. 1b,c biphasic and epithelioid samples had significantly higher fractal dimension and lower lacunarity compared to hyperplastic/benign tissue (p < 0.0001). Although the difference between biphasic and epithelioid tissue was not found to be statistically significant, epithelioid tissue tended to have a slighter higher fractal dimension and lower lacunarity than biphasic tissue. These results suggest that fractal dimension and lacunarity analysis maybe a useful and rapid method to differentiate benign and malignant tissues in malignant mesothelioma. No significant variation was detected between duplicate samples taken from the same tumor (Supplemental Fig. 1). No correlation was found between age, gender or survival with fractal dimension or lacunarity (Data not shown). The diagnostic potential of fractal dimension and lacunarity was determined by receiver operating characteristic (ROC) analysis. The cases of biphasic and epithelioid mesothelioma were differentiated from benign hyperplasia. Analysis indicates fractal dimension and lacunarity as excellent predictors of mesothelioma histology. For fractal dimension the areas under the curve (AUC) for cases of biphasic or epithelioid mesothelioma were 96.97% and 91.84% respectively. For lacunarity the AUC’s for biphasic or epithelioid mesothelioma were 97.06% and 90.00% respectively. Therefore both fractal dimension and lacunarity demonstrated high accuracy as predictors of mesothelioma. ROC curves, sensitivity and specificity results are included in supplemental Figs 2 and 3.

### Mitochondrial morphology in mesothelioma

**Figure 2:**
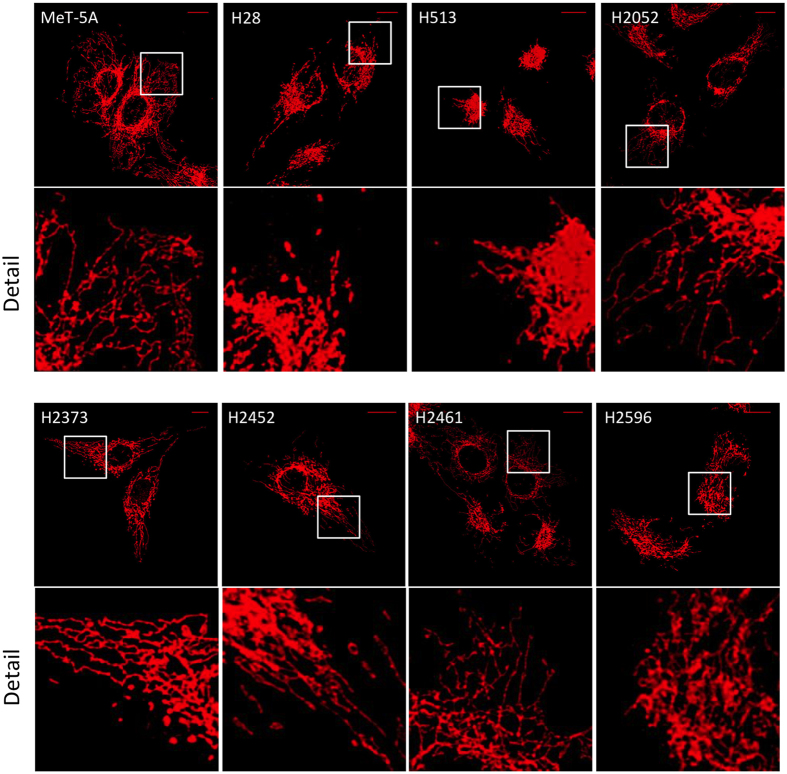
Mitochondrial morphology in malignant mesothelioma cell lines. Mesothelioma cell lines were fixed in 4%paraformaldehyde and stained with TOM20 and Alexa647. Confocal Z-stack images were captured using a Leica SP5 II and a maximum projection image was generated using ImageJ. Scale bar shown is 10 μm. Detailed image from highlighted area is shown for each cell type.

**Figure 3:**
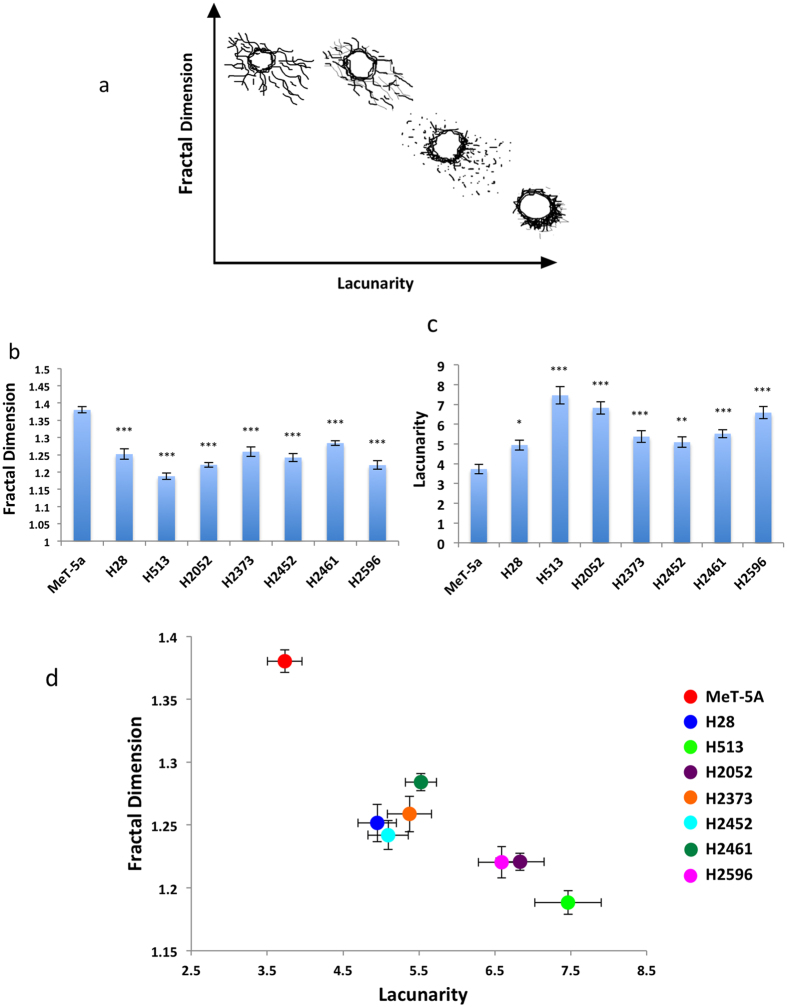
Fractal dimension and lacunarity of mitochondria. (**a**)Examples of typical mitochondrial morphologies obsereved in mesothelial cell lines classified via fractal dimension and lacunarity. Maximum projection of Z-stack image series of TOM20 mitochondrial marker stain was used to quantify (**b**) mitochondrial fractal dimensions and (**c**) lacunarity. Images were converted to grayscale and analysed using the FracLac plugin from ImageJ. The entire mitochondria of each cell was analyzed. Data are presented as mean ± SEM, significance was calculated using an ANOVA with Tukey post test (*p < 0.05, **p < 0.005, ***p < 0.0001) n ≥ 40 for each cell type (**c**). (**d**) Profile of mesothelioma mitochondrial morphologies classified via fractal dimension and lacunarity.

We then applied the fractal dimension and lacunarity analysis to mitochondrial morphology in a panel of mesothelioma cell lines. Staining with the mitochondrial marker TOM20 revealed a spectrum of mitochondrial morphologies in the mesothelioma cell lines (Fig. 2). MeT-5A cells had reticulated and elongated mitochondria distributed throughout the cytoplasm. The mesothelioma cell lines showed a range of alterations, from H28 in which the mitochondria were much more fragmented but still distributed throughout the cytosol, to H513 which had some elongated mitochondria but they were almost exclusively perinuclear. H2052 and H2596 cells had a high level of reticulated mitochondria but with a restricted distribution around the nucleus. In order to quantify these different complex mitochondrial morphologies we calculated the mitochondrial fractal dimension and lacunarity of each cell type using the ImageJ plugin FracLac^34^. A minimum of 40 cells were analyzed for each cell type and the entire mitochondrial network was analyzed for each cell. Figure 3a illustrate the general relationship between fractal dimension, lacunarity and the types of mitochondrial morphologies observed in the mesothelioma cells. The mesothelioma cell lines all possessed significantly decreased fractal dimensions compared to control MeT-5A cells, with H513 and H2596 showing the largest changes (Fig. 3b). Conversely, lacunarity was increased in the mesothelioma cell lines compared to MeT-5A controls (Fig. 3c). Again H513 and H2596 showed the most significant changes compared to MeT-5A. Fractal dimension and lacunarity takes into account the fission/fusion state of the mitochondria, the diameter of the mitochondrial tubules and the distribution within the cell. Figure 3d shows a morphological profile of the mesothelioma cells based on fractal dimension and lacunarity. We can see that the control MeT-5A cells have a different profile to the mesothelioma cells. H513 is at the opposite end of the spectrum to MeT-5A, its mitochondria are almost completely perinuclear showing limited elongation (Fig. 3d).

### Altered expression of mitochondrial proteins in malignant mesothelioma cell lines

**Figure 4:**
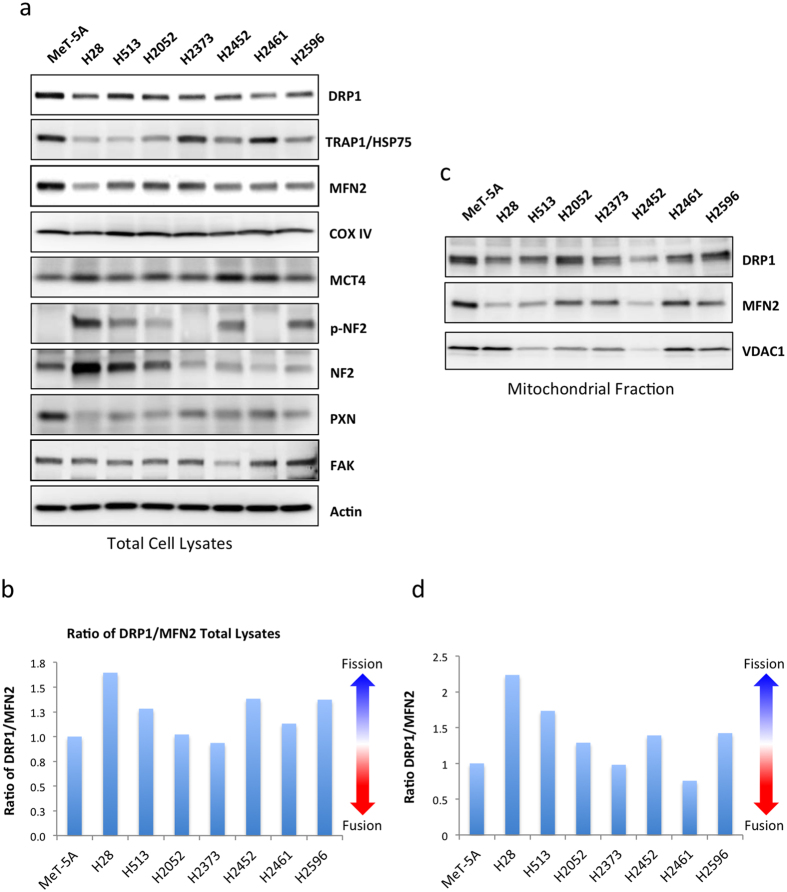
Altered expression of mitochondrial proteins in malignant mesothelioma cell lines. (**a**) Representative immunoblots showing expression of mitochondrial and focal adhesion marker proteins in a panel of mesothelioma cell lines and a control transformed but non-tumorgenic cell line, MeT-5A. (**b**) Ratio of DRP1 to MFN2 expression in total cell lysates as measured via densitometry (**c**) Representative immunoblots showing expression of DRP1, MFN2 and VDAC in the mitochondrial fraction of mesothelioma cell lysates (**d**) Ratio of DRP1 to MFN2 expression in the mitochondrial fraction of mesothelioma cell lysates as measured via densitometry. 20 μg of protein was loaded per sample. Results are expressed as mean ± the standard deviation of 3 independent measurements.

Mitochondrial dynamics are regulated in part *via* the relative expression of fission and fusion proteins including DRP1, MFN2, OPA1 and FIS1^35^,^36^. To analyze the expression of mitochondrial dynamics proteins in mesothelioma we performed immunoblotting on the panel of mesothelioma cell lines (Fig. 4a). There was a decrease in total DRP1 and MFN2 in mesothelioma cell lines compared to the MeT-5A mesothelium control cells, suggestive of decreased fission/fusion cycling in these cells. However there was also a shift in the relative levels of these proteins in the mesothelioma cell lines towards higher expression of the fission regulator DRP1 compared to MFN2, the fusion regulator (Fig. 2b). This could suggest a higher relative rate of fission in these cells, which would result in a more fragmented mitochondrial network. The expression of TRAP1, an HSP75-like protein involved in the recruitment of DRP1 to the mitochondria was also altered in mesothelioma, although no clear pattern emerged^37^,^38^. Expression of COX IV a mitochondrial marker protein was similar in all cell lines indicating no significant changes in total mitochondrial content^39^. MCT4, which facilitates lactate export from cells, is associated with a glycolytic phenotype^40^,^41^. MCT4 was increased in mesothelioma cell lines compared to control MeT-5A cells. This increase was most evident in cell lines expressing low levels of total NF-2 or those with phosphorylated, inactive NF-2, namely H28 and H2461 (Fig. 4a).

We then examined the levels of DRP1 and MFN2 localized to the mitochondria. Mitochondrial fractions were isolated from each cell line and immunoblotted. Similarly to the trend observed in total cell lysates, DRP1 levels were increased relative to MFN2 in mitochondrial fraction of the mesothelioma cells lines (Fig. 2c,d). Protein levels were normalized using expression of the mitochondrial marker protein VDAC1.

### Mitochondrial and glycolytic function in mesothelioma

**Figure 5:**
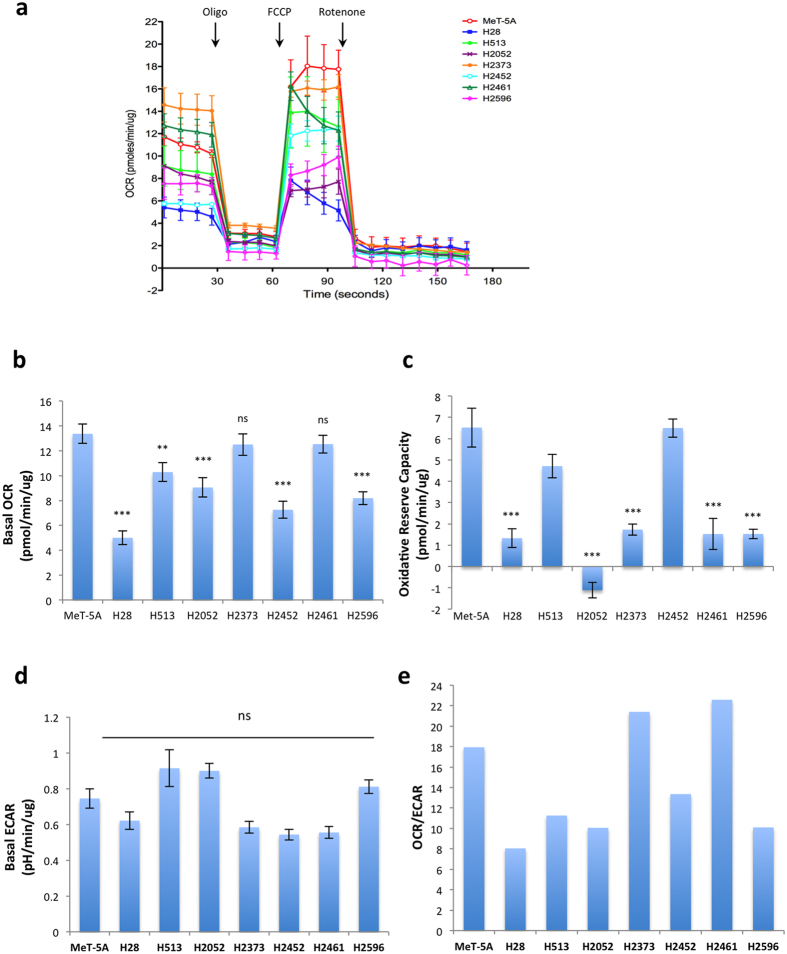
Mitochondrial oxidative and glycolytic activity in malignant mesothelioma cell lines. Oxygen consumption rate (OCR), an indicator of mitochondrial oxidative phosphorylation, and extracellular acidification rate (ECAR) an indicator of glycolytic activity measured over time via Seahorse extracellular flux analyzer. The mitochondrial stress test was used to obtain the bioenergetic parameters. (**a**) shows an example of one mitochondrial stress assay, arrows indicate the addition of 0.5 μM oligomycin A (oligo), 0.5 μM FCCP, and 1 μM Rotenone. (**b**) Basal mitochondrial OCR, (**c**) mitochondrial respiratory reserve capacity, obtained by subtracting basal OCR from maximum OCR, (**d**) basal ECAR (**e**) ratio of OCR to ECAR. Results are shown as the mean ± SEM of 5 independent experiments and analyzed via ANOVA with Tukey’s post-test (*p < 0.05, **p < 0.005, ***p < 0.0001).

**Figure 6:**
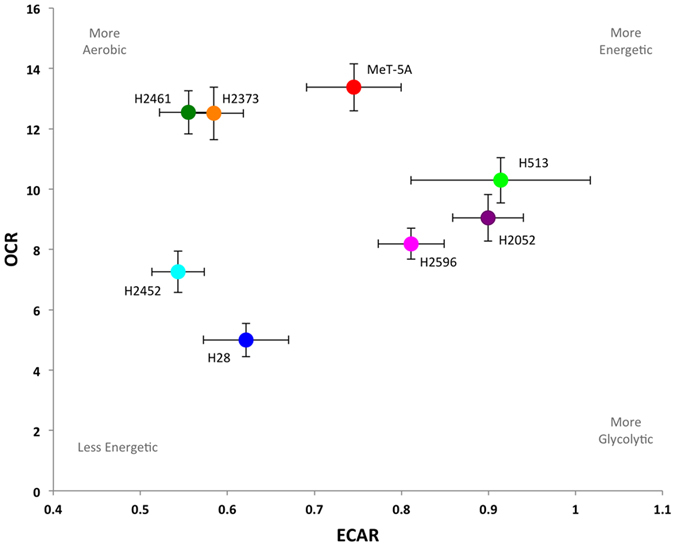
Bioenergetic profile of malignant mesothelioma cell lines. Plot of basal ECAR and OCR levels in mesothelioma and control cell lines, mean values ± SEM are shown. From the plot we can easily see that H28 and H2452 are the least energetic cell lines, while MeT-5A and H513 have relatively high levels of both oxidative phosphorylation and glycolysis and are therefore more energetic. H2461 and H2373 are more aerobic, while H2461 and H2596 are more glycolytic.

Mitochondrial morphology is also thought to be an indicator of mitochondrial activity. Fusion or elongation of mitochondria is associated with optimization of mitochondria function and preservation of mitochondrial membrane potential, while fission is associated with removal of damaged mitochondrial components and may be an indicator of decreased mitochondrial function^26^. Given the varied mitochondrial morphologies and altered expression of mitochondrial dynamics associated proteins in mesothelioma we investigated the bioenergetic profile of these cells using a mitochondrial stress test. OCR was measured as an indicator of oxidative phosphorylation while glycolysis was assessed via the ECAR^32^. An example of a typical mitochondrial stress test in shown in Fig. 5a. Basal OCR was significantly lower in multiple mesothelioma cell lines (p < 0.001 for H28, H2052, H2452, and H2596) compared to control MeT-5A cells (Fig. 5b). Two mesothelioma cell lines, H2373 and H2461, had no change compared to control MeT-5A cells. The majority of mesothelioma cells, with the exception of H513 and H2452, also had significantly reduced levels of reserve oxidative respiratory capacity (calculated as the difference between max OCR stimulated by FCCP treatment and basal OCR levels) (Fig. 5c)^32^. Basal ECAR, a measure of glycolytic activity was almost unchanged in the mesothelioma cell lines compared to MeT-5A (Fig. 5d). H513 and H2052 exhibited slightly higher ECAR but these did not reach statistical significance. Similarly, the lower ECAR observed in H2372, H2452 and H2461 did not reach significance. Fig. 5e shows the ratios of OCR to ECAR in these cells lines. A lower ratio indicates cells are more dependent on glycolysis compared to oxidative phosphorylation^42^. The majority of the mesothelioma cell lines tested show a decrease in this ratio indicating an increased dependence on glycolysis compared to Met-5A cells (H28, H513, H2052, H2452, H2596). Two of the cell lines tested (H2373 and H2461) had a higher OCR/ECAR ratio compared to control MeT-5A cells. Figure 6, places the cell lines on the OCR-ECAR plane and gives us a snapshot of the basal bioenergetic profile of these cells and how they relate to the control MeT-5A cells. We can see that MeT-5A cells have relatively high oxidative phosphorylation activity, while H28 and H2452 have lower levels of both oxidative phosphorylation and glycolysis and can therefore be considered as a low energy phenotype. Incidentally these two cell lines had the lowest proliferation rate of the cell lines tested (data not shown).

### Cytotoxicity of mitochondrial inhibitors in malignant mesothelioma

**Figure 7:**
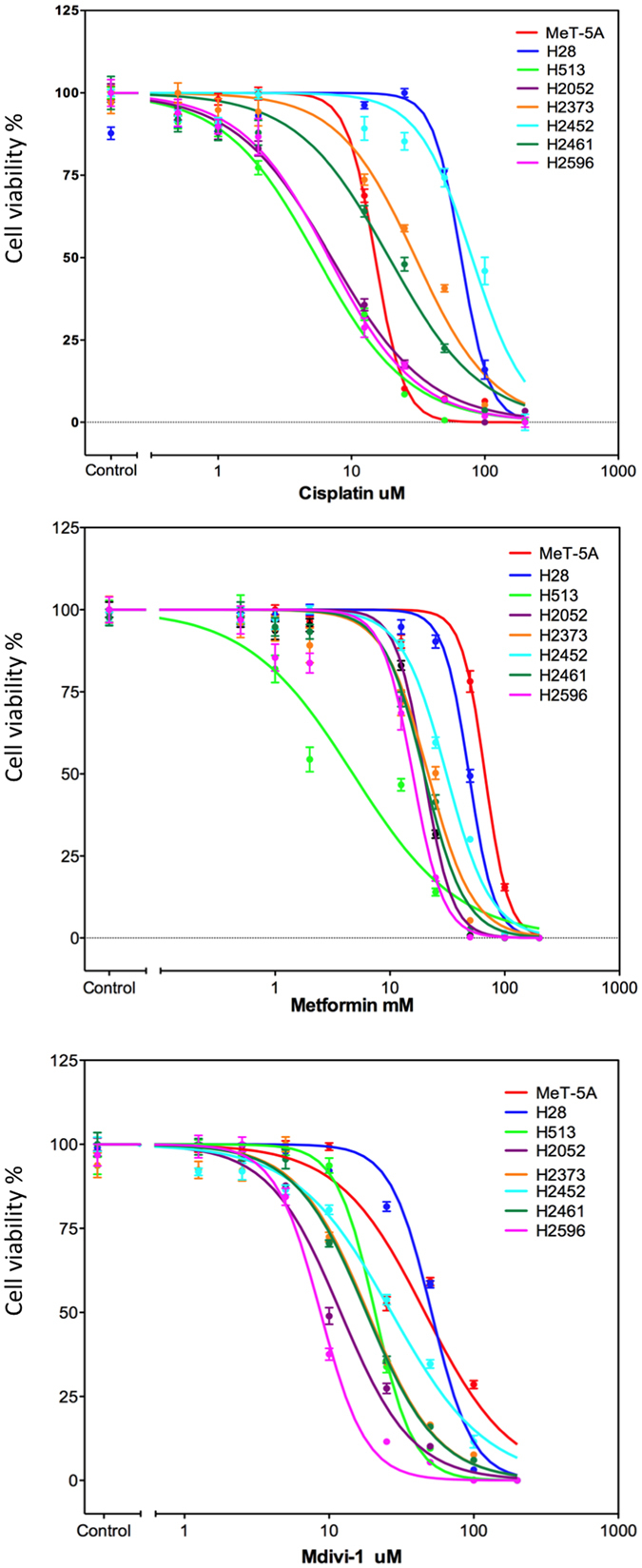
Cytotoxicity of mesothelioma cell lines to cisplatin, metformin and mdivi-1. Mesothelioma and control cells in 1% serum media were treated with increasing concentrations of (**a**) Cisplatin, (**b**) Metformin, or (**c**) mdivi-1 as indicated for 72 hrs and cell viability was then assessed via Calcein-AM uptake as described. The results were normalized as a percentage of the untreated controls and analyzed via a sigmoidal dose response curve using Prism software (version 5.0) to calculate the EC50 value for each drug, n = 6 for each concentration shown ± the standard error of the mean.

Next we examined the cytotoxicity of two different mitochondrial targeted inhibitors, metformin and mdivi-1, and cisplatin, a standard chemotherapeutic agent in mesothelioma cell lines^1^. The first inhibitor metformin, is reported to inhibit complex I of the electron transport chain and is under investigation for its potential as a cancer therapeutic and preventative agent^43^. The second inhibitor, mdivi-1 is a specific inhibitor of DRP-1, a key regulator of mitochondrial fission^44^. Cells were treated with metformin, mdivi-1, cisplatin or vehicle control at the indicated concentrations for 72 hrs (Fig. 7). The EC50 for each drug was then calculated and is shown in Table 1. In the case of cisplatin, H2452 and H28 cells lines showed the highest levels of resistance to cisplatin treatment, with EC50 values of 80.13 μM and 66.63 μM respectively. H513 and H2596 were the most sensitive (EC50 5.5 μM and 6.769 μM). The control cell line MeT-5A was also sensitive to cisplatin toxicity compared to the mesothelioma cell lines with an EC50 of 15.1 μM. However when cells were treated with the mitochondria targeted therapies a different profile emerged. MeT-5A showed the highest resistance to metformin (EC50 67.28 mM) of all cell lines tested. H28 and H2452 were also somewhat resistant to metformin treatment. These two cell lines also had the slowest proliferation rate of the cell lines tested (data not shown). H513 and H2596 were the most sensitive cell lines (EC50 4.762 mM and 15.8 mM) being 14 and 4 times more sensitive than MeT-5A. Cells treated with mdivi-1 showed a similar trend to the metformin treatment. H28 and MeT-5A were the most resistant to treatment (EC50 51.11 μM and 46.0 μM) while H2596 was the most sensitive (EC50 8.722 μM).

**Figure 8:**
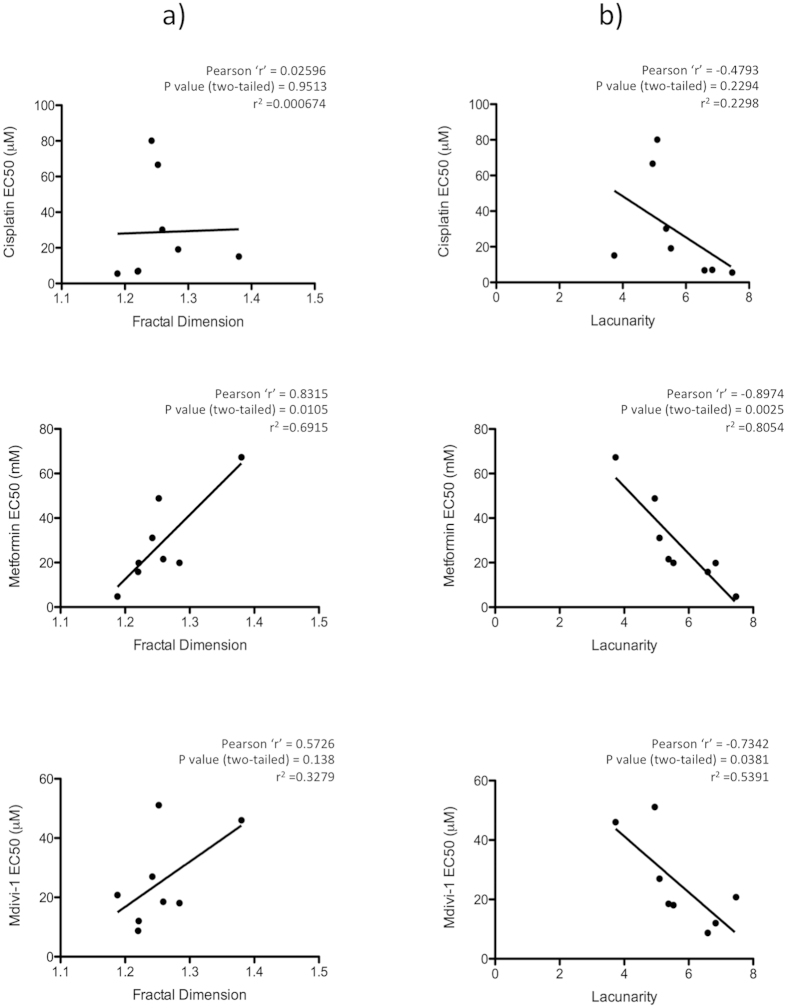
Correlation between EC50 and mitochondrial morphology. Scatter plot sowing correlation between (**a**) Fractal dimension and (**b**) Lacunarity and inhibitor sensitivities (EC50 values) for cisplatin, metformin and mdivi-1. Significant correlation is observed between fractal dimension and metformin EC50 and between lacunarity and metformin and mdivi-1 EC50. Pearson correlation coefficient was calculated for each data pair as indicated.

Given the spectrum of mitochondrial morphologies and bioenergetic activity observed in the mesothelioma cells we investigated whether any of these factors correlated with the inhibitor sensitivities. We plotted EC50 values for each cell line and inhibitor against basal OCR, ECAR, mitochondrial fractal dimension and lacunarity and calculated the Pearson correlation coefficient. Mitochondrial fractal dimension showed statistically significant correlation with metformin sensitivity (r = 0.8315, *p* = 0.0105) and a slight but not significant correlation with Mdivi-1 (r = 0.5726, *p* = 0.138) but did not correlate with cisplatin sensitivity (r = 0.02596, *p* = 0.915) (Fig. 8a). Mitochondrial lacunarity demonstrated significant inverse correlation with both metformin (r = −0.8974, *p* = 0.0025) and mdivi-1 (r = −0.7342, *p* = 0.0381), but no correlation with cisplatin sensitivity (r = −0.4793, *p* = 0.2294) (Fig. 8b). ECAR demonstrated an inverse correlation with cisplatin sensitivity (r = −0.715, *p* = 0.0462) but did not correlate with either of the mitochondrial inhibitors (Supplemental data Fig. 4). OCR did not correlate with any of the inhibitors (Supplemental data Fig. 4).

## Discussion

In this study, we have applied the metrics of fractal dimension and lacunarity to the quantification of mesothelioma tumor tissue and mitochondrial morphologies. These measurements were successfully used to differentiate between hyperplastic and malignant mesothelial tissues and to classify various mitochondrial morphologies in MM cell lines. The expression of mitochondrial fission and fusion regulatory proteins (DRP1 and MFN2 being the main regulators) was altered in MM, likely contributing to the various morphologies. The panel of MM cell lines examined displayed a spectrum of bioenergetic profiles, ranging from more oxidative (H2461), to more glycolytic (H28) and with decreased oxidative reserve capacities. MM cell lines exhibited increased sensitivity to mitochondrial-targeted inhibitors, be it metformin (complex 1 inhibitor) or mdivi-1 (DRP1 inhibitor) compared to control MeT-5A cells. Finally we report that mitochondrial morphology measured via fractal dimension or lacunarity correlated with mitochondrial inhibitor (metformin, mdivi-1) sensitivity, whereas OCR or ECAR did not show any correlation (Fig. 8a,b and Supplemental Fig. 4a,b).

Tissue and cell morphologies cannot always be readily and completely quantified using standard Euclidian metrics. For example, automated quantification of mitochondrial morphology within the cells is challenging and often restricted to simple measurements of length or volume^45^. Other visual methods of classification are heavily dependent on subjective interpretation^28^. Both of these approaches can be time-consuming and often do not take into account subtle differences in mitochondrial structure and the mitochondrial distribution within the cell. The same is also true of tissue measurements. Therefore we have adopted a more objective and complete quantification method for complex patterns and shapes such as those found in tumor tissues or the mitochondrial network using fractal dimension and lacunarity measurements^18^,^20^,^21^,^22^. Fractal dimension analysis allows us to quantify the inherent self-similarity in a structure or image. It tells us how the image changes based on the scale used to observe said image^15^. Lacunarity relates to texture, it quantifies the distribution and size of gaps within an image^23^. Fractal dimension and lacunarity have previously been utilized to quantify complex morphologies and patterns in a range of biological settings including cell shape, chromatin texture, retinal angiogenesis, neuronal growth and physiological measurements^33^,^46^,^47^,^48^,^49^,^50^. We observed a significant difference in the fractal dimension and lacunarity of hyperplastic compared to malignant mesothelial tissue samples (Fig. 1b,c). ROC analysis indicates fractal dimension and lacunarity as excellent predictors of mesothelioma histology (Supplemental Figs 2 and 3). Further analysis of larger numbers of tissue samples including the sarcomatoid tumors will be necessary to assess if these measurements would also be useful in discriminating between mesothelioma subtypes.

Cell metabolism and mitochondrial function is a crucial facet of MM and an emerging therapeutic target^51^,^52^. We undertook an investigation of multiple aspects of the mitochondria in MM including morphology, protein expression and bioenergetic function. Fractal dimension and lacunarity analysis of mitochondrial morphologies revealed significant differences in MM cell lines (Fig. 3b,c). Fractal dimension was correlated with sensitivity to metformin inhibition, while lacunarity was inversely correlated with sensitivity to both metformin and mdivi-1 (Fig. 8). Mitochondrial oxidative phosphorylation measured via OCR did not correlate with any of the inhibitors tested (Supplemental Fig. 4). These results suggest that mitochondrial morphology could be an important predictor of mitochondrial inhibitor sensitivity, even more so than bioenergetic function. Expression of multiple mitochondrial dynamics associated proteins are altered in MM. Expression of both DRP1, a fission regulator and MFN2, a fusion regulator are decreased in MM compared to control MeT-5A cells (Fig. 4a) other fission and fusion regulators FIS1, OPA1 also show differential expression in these cells (Supplemental Fig. 5). This may indicate a decrease in the overall rate of fission-fusion cycling in these cells. When we examined the ratio between DRP1 and MFN2, it indicates that in MM there is an increase in the relative level of DRP1 compared to MFN2, particularly in the mitochondrial fraction (Fig. 4b–d). This would indicate a pro-fission mitochondrial phenotype in these cells. Increased fission is associated with decreased mitochondrial activity^26^. We have previously reported that lung cancer cells have decreased levels of mitochondrial networking and increased fission^44^. An overall decrease in the rate of fission-fusion cycling could mean the mitochondria are slower to clear damaged mitochondrial proteins and may be less effective in maintaining the mitochondrial membrane potential. This would result in decreased mitochondrial performance and increased cell death. This could explain the increased sensitivity of MM cells to mitochondrial-targeted inhibitors (Table 1).

Comparing the bioenergetic profile (Fig. 6) with the fractal analysis (Fig. 3d) we observed some clustering of certain cell types in both graphs. H2373 and H2461 both have relatively high levels of OCR and high fractal dimension/low lacunarity, while H28, H2452 exhibit low metabolic activity and relatively low fractal dimension and lacunarity. However we did not detect any significant correlation between mitochondrial morphology measurements (fractal dimension, lacunarity) with OCR or ECAR. The lack of a definitive association between morphology and metabolic activity is not completely unexpected as other factors apart from morphology can affect mitochondrial activity. Substrate availability, reactive oxygen species and multiple signaling pathways can all effect mitochondrial function and activity^53^.

While the alteration in mitochondrial morphology may in part be due be to altered activity and expression of mitochondrial fission and fusion regulators it may also reflect changes in mitochondrial transport and localization in the cytoplasm. Mitochondria are transported mainly via the microtubule network while the actin cytoskeleton may be involved in mitochondrial anchoring and in regulating fission^54^,^55^,^56^. Regulation of the actin cytoskeleton is quite complex and is influenced by a plethora of signals, particularly during cell migration^57^. The link between metabolism and/or the mitochondria and cell migration is an area of growing research interest^35^,^58^,^59^,^60^. Together with the actin cytoskeleton, focal adhesion proteins are another crucial regulator of cell migration. Recently, we have shown that focal adhesion protein paxillin can influence the mitochondrial architecture^61^. For example, when the mutant A127T paxillin protein, which stimulates focal adhesion formation, is introduced into cells the mitochondrial network appears swollen and more reticulated. This indicates an alteration in the mitochondrial fission-fusion cycling within the cell. In this case it appears that paxillin is downregulated in mesothelioma compared to control cells, which may be indicative of altered migration and focal adhesion regulation in these cells (Fig. 4a). FAK, another crucial regulator of cell migration and emerging therapeutic target in mesothelioma may also be regulated via metabolic stress^58^. However we did not observe significant alteration in FAK expression in the mesothelioma cell lines, with the exception of H2452 where it was down regulated. Given that mesothelioma cells display a range of mitochondrial morphologies a more systemic investigation of the reciprocal regulation of the cytoskeleton and mitochondrial dynamics and functionality in cell migration is now warranted. It is also possible that differential gene alterations (such as loss or mutation of NF2 or BAP1) can also contribute to metabolic changes and altered mitochondrial morphologies in mesothelioma and should be more thoroughly investigated.

The use of metformin as a cancer preventative and therapeutic agent is an attractive but still largely unproven proposition. While initial observational reports from diabetic patients indicated that metformin treatment could be an effective cancer prevention strategy more formal studies have yet to confirm this^62^,^63^,^64^,^65^. Further its effectiveness as a cancer therapeutic remains unclear with conflicting reports and no consensus on its use or effects^51^. Our data indicate that mitochondrial morphology may be an informative metric in the prediction of metformin and even mitochondrial inhibitor sensitivity. Previous reports have indicated that the mitochondria and cell metabolism may be a viable therapeutic target in MM. Inhibition or silencing of peroxirodoxin-3, a mitochondrial oxidoreductase, led to disrupted mitochondrial bioenergetics, cell cycle arrest and reduced MM tumor volumes *in vivo*^52^. Here we have also shown the effectiveness of targeting mitochondrial dynamics to inhibit cell viability in MM. We have previously shown that mdivi-1 inhibits cell proliferation *in vitro* and decreased tumor volumes *in vivo* in lung cancer^44^. The data shown here indicate that it may also be effective against MM tumor growth.

In conclusion we have shown that mitochondrial morphology is altered in mesothelioma. This is reflected in altered expression of mitochondrial dynamics regulatory proteins and in altered cell bioenergetics. Classification of mitochondria via fractal dimension and lacunarity is a rapid, robust and objective method to quantify subtle changes in morphology and may be a useful indicator of sensitivity to mitochondrial-targeted inhibition. Going forward, we believe that the mitochondria is a viable target in mesothelioma and that mitochondrial morphology may be a promising predictor of therapeutic response.

## Materials and Methods

### Cell Culture and Reagents

MeT-5A control transformed non-tumorigenic mesothelial cells (ATCC) were maintained in Medium 199 containing 10% fetal bovine serum (Sigma), 1.5 g/L sodium bicarbonate (Corning), 3.3 nM epidermal growth factor, 400 nM hydrocortisone, 870 nM zinc-free bovine insulin (Lonza), 20 mM HEPES (Sigma), 3.87 μg/L selenious acid, trace elements B liquid (MediaTech) used at 1,000 dilution. Mesothelioma cell lines H28, H513, H2052, H2461 (Epithelioid) and H2373, H2596 (Sarcomatoid) and H2452 (Biphasic) (ATCC) were maintained in RPMI-1640 (Corning), 10% fetal bovine serum (Sigma) as described previously. Metformin, Cisplatin, and mdivi-1 were obtained from Sigma.

### Cell metabolism

A Seahorse Bioscience XF24 Extracellular Flux Analyzer was used to measure OCR and ECAR. Control and mesothelioma cell lines were maintained in normal complete growth media and seeded onto a gelatin coated 24-well XF Flux analyzer assay plate at 80,000 cells/well 24 hrs prior to assay. Cells were switched to serum free XF assay media (Seahorse Biosciences) with 25 mM glucose, 1 mM sodium pyruvate, 2 mM glutamine (Corning) and placed in a CO_2_-free incubator at least 1 hour prior to start of assay. A Seahorse XF24 Analyzer was then used to measure the cellular bioenergetic profile. Each cycle included 3 min of mixing, a 2 min wait and finally measurement over 2 mins. Four measurements were obtained at baseline and following injection of oligomycin (1 μM; Sigma), FCCP (1 μM; Sigma), and rotenone (1 μM; Sigma). Measurements were normalized to total protein content per well using the Bradford protein assay (Bio-Rad).

### Immunoblotting

Cells were rinsed with PBS and scraped in ice-cold RIPA lysis buffer (Boston BioProducts) and centrifuged at 8,000 rpm for 5 min at 4 °C. A Coomassie Bradford protein assay (Thermo Fisher) was performed and equal amounts of protein were combined with 4x reducing Laemmli sample buffer (Boston Bioproducts) prior to loading on to SDS-page gel. 20 μg of protein was loaded for each sample and separated by SDS–PAGE, and immunoblotted with antibodies to DRP1 (Novus Biologicals), TRAP1, MFN2, MCT4, VDAC1 (Santa Cruz Biotechnology) NF2, phospho-NF2 (Cell Signaling Technology), FAK (Millipore), Paxillin (Invitrogen) Actin (Sigma). After incubation with peroxidase-conjugated secondary antibodies, the signals were visualized by Clarity**™** enhanced chemiluminescence (Bio-Rad) according to the manufacturer’s instruction. Protein band intensity was quantified by ImageJ software.

### Isolation of Mitochondrial Fraction

The mitochondrial fraction was isolated according to a protocol adapted from Wheaton *et al*.^66^. Briefly, cells were rinsed in ice-cold PBS and scraped in mitochondrial isolation buffer (250 mM Sucrose, 50 mM Tris-HCl pH7.4, 0.1 mM EGTA, 5 mMMgCl with protease inhibitors). Cells were disrupted by 30 strokes with a Dounce homogenizer (15 strokes with each pestle) and 5 expulsions through a 28-gauge needle. The lysates were centrifuged at 1000 rpm for 10 min to remove nuclei, followed by centrifugation at 11,000 rpm for 20 min to pellet mitochondria. The mitochondrial pellet was then solubilized in RIPA buffer and the protein concentration determined via Bradford assay (Bio-Rad). Samples were then processed for immunoblotting as described earlier.

### Tissue Microarray

The Institutional Review Board at The University of Chicago (Chicago, IL) approved all of the human subjects research performed in this study. Written informed consent was obtained from all patients involved in the study. All methods were carried out in accordance with the respective approved protocol. Duplicate samples from fifty-seven MM tumors including 36 epithelioid, and 20 biphasic were processed into a tissue microarray (TMA) under an institutional review board approved protocol as previously described^67^. Patient and tumor characteristics are included in Table 2. Control tissues included 15 benign hyperplastic mesothelial samples in duplicate. The TMA was built using the ATA-27 Arrayer from Beecher Instruments. In brief, tissue cores (1-mm punch) from biopsied tissue samples were precisely organized into a grid and embedded in paraffin. The paraffin block was cut and the TMA was processed for H&E staining. Pathologic diagnosis in these cases was reviewed by at least two experienced pathologists. The H&E stained TMA was scanned using a 3D- Histech Pannoramic SCAN whole slide scanner.

### Immunofluorescence

Cells were cultured for 24 hrs on glass coverslips and fixed in 4% paraformaldehyde for 10 mins at 37 °C. They were permeabilized in 0.2% Triton PBS for 2 mins and blocked in 5%BSA PBS. Slides were incubated with primary antibody TOM20 (Santa Cruz Biotechnology) for 30 mins at RT followed by Alexa-647 conjugated secondary anti-Rabbit. Slides were then mounted using Prolog Gold anti-Fade mounting media (Life Technologies). Slides were then analyzed and imaged using Leica SP5 II STED-CW superresolution Laser Scanning Confocal.

### Fractal dimension and lacunarity analysis

For analysis of the TMA histological samples 15x images of each core were selected from the whole slide scanned images. These images were converted to grayscale and fractal dimension and lacunarity was analyzed using the ImageJ plugin FracLac^34^. A box-counting grayscale differential analysis returns an intensity fractal dimension based on the difference in pixel intensity in each box. 12 different grid positions were used and the box sizes ranged from a minimum of 2 × 2 pixels and increased until they reached a maximum size of 45% of the selected image area. The estimated fractal dimension is calculated for each grid position from the regression line of a log-log plot of intensity versus box size. The average of these estimates is then calculated as the fractal dimension. Lacunarity was calculated based on the number of positive pixels in each box at the different box sizes.

Immunofluorescent stacks of TOM20-Alexa-647 stained cells were converted to maximum projection images using ImageJ. Background intensity was analyzed for all images and 1000 was subtracted from each image to ensure a zero background prior to fractal dimension analysis. The entire mitochondrial network of each individual cell was selected and saved as a separate image. The FracLac ImageJ plug-in was then used to measure the fractal dimension and lacunarity of each image as described.

### Cytotoxicity assays

To determine specific cytotoxicity, we used cell-permeable Calcein-AM (Santa Cruz). The hydrolysis of Calcein-AM by intracellular esterases produces Calcein, a hydrophilic, strongly fluorescent compound that is well-retained in the cell cytoplasm. Cells were seeded in black walled 96-well plates and allowed to adhere in normal growth media for 24 hrs. Cells were then washed once in PBS and the test compounds were added in 1% serum media at the indicated concentrations for 72 hrs. The media was then removed and replaced with 100 ul of phenol red-free optimem containing 2 μM Calcein-AM and incubated for a further 30 mins. Fluorescence was then measured at 485/535 nm using a Bio-Tek Synergy multi-detection microplate reader.

### Statistical analysis

The Graph Pad Prism 5.0 software package was used for the statistical analysis. One-way ANOVA with Tukey multiple comparison post-test was used as appropriate. EC50 values were calculated by fitting a sigmoidal ‘four parameter logistic’ curve to the normalized log-transformed data. Pearson ‘r’ coefficient was calculated to determine correlation. A *p* value of less than 0.05 was considered significant. ROC analysis was performed using the STATA12 Data Analysis and Statistical Software.

## Additional Information

**How to cite this article**: Lennon, F. E. *et al*. Unique fractal evaluation and therapeutic implications of mitochondrial morphology in malignant mesothelioma. *Sci. Rep*. **6**, 24578; doi: 10.1038/srep24578 (2016).

## Supplementary Material

Supplementary Information

## Figures and Tables

**Table 1:** EC50 values for mesothelioma cell lines.

	MeT-5A	H28	H513	H2052	H2373	H2452	H2461	H2596
Cisplatin (uM)	15.11 ± 1.021	66.63 ± 1.034	5.5 ± 1.054	7.078 ± 1.051	30.17 ± 1.057	80.13 ± 1.055	19.16 ± 1.077	6.769 ± 1.078
Metformin (mM)	67.28 ± 1.025	48.82 ± 1.018	4.762 ± 1.135	19.86 ± 1.023	21.55 ± 1.057	31.07 ± 1.027	19.88 ± 1.034	15.8 ± 1.05
Mdivi-1 (uM)	46 ± 1.07	51.11 ± 1.028	20.78 ± 1.025	12.03 ± 1.038	18.5 ± 1.047	26.98 ± 1.043	18.08 ± 1.035	8.722 ± 1.029

**Table 2:** Patient and mesothelioma tumor characteristics.

Gender	Male	41
	Female	13
Age (Years)	Mean	69
	Range	46–91
Survival (Months)	Mean	19
	Range	1–105
Tumor Characteristics		No. of cases
Biphasic		20
Epithelioid		36
pT Status	T1	1
	T2	2
	T3	7
	T4	9
pN Status	N0	8
	N1-3	11
pM Status	Mx	4
	M1	–

Numbers may not sum to total N due to missing data.
