# Supplementary material for: Unique fractal evaluation and therapeutic implications of mitochondrial morphology in malignant mesothelioma
Source: Sci Rep. 2016 Apr 15;6:24578. doi: 10.1038/srep24578 (PMC4832330; doi:10.1038/srep24578)

Unique fractal evaluation and therapeutic implications of mitochondrial morphology in malignant mesothelioma

Frances E Lennon, Gianguido C Cianci, Rajani Kanteti, Jacob J Riehm, Qudsia Arif, Valeriy A Poroyko, Eitan Lupovitch, Wickii Vigneswaran, Aliya Husain, Phetcharat Chen, James K Liao, Martin Sattler, Hedy L Kindler, Ravi Salgia

Supplemental Data

Supplemental Figure 1. Fractal Dimension and Lacunarity analysis of duplicate tumor samples.

Fractal dimension and lacunarity were measured for duplicate samples from control hyperplasia/benign, biphasic and epithelioid mesothelioma samples as technical replicates. Results were analyzed via two-way repeated measures ANOVA. No significant variation was detected between the duplicate samples in any of the subtypes.

Supplemental Figure 2. ROC Analysis of Fractal dimension classifer

ROC analysis was carried out on fractal dimension for a)biphasic and b) epithelioid mesothelioma samples.

Supplemental Figure 3. ROC Analysis of Lacunarity classifer.

ROC analysis was carried out on lacunarity for a)biphasic and b) epithelioid mesothelioma samples.

Supplemental Figure 4. Scatter plots of OCR and ECAR with inhibitor EC50 values.

Scatter plots of a)OCR, b)ECAR with EC50 values for cisplatin, metformin and mdivi-1. Calculation of Pearson correlation coeffient ‘r’ did not detect any significant correlation with the exception of ECAR and cisplatin where r= -0.715, *p*=0.0462

Supplemental Figure 5. Expression of mitochondrial and metabolic regulatory proteins in malignant mesothelioma.

Representative immunoblots showing expression of indicated mitochondrial and metabolic regulatory proteins in a panel of mesothelioma cell lines and a control transformed but non-tumorgenic cell line, MeT-5A.

Supplemental Figure 1. . Fractal Dimension and Lacunarity analysis of duplicate tumor samples

a

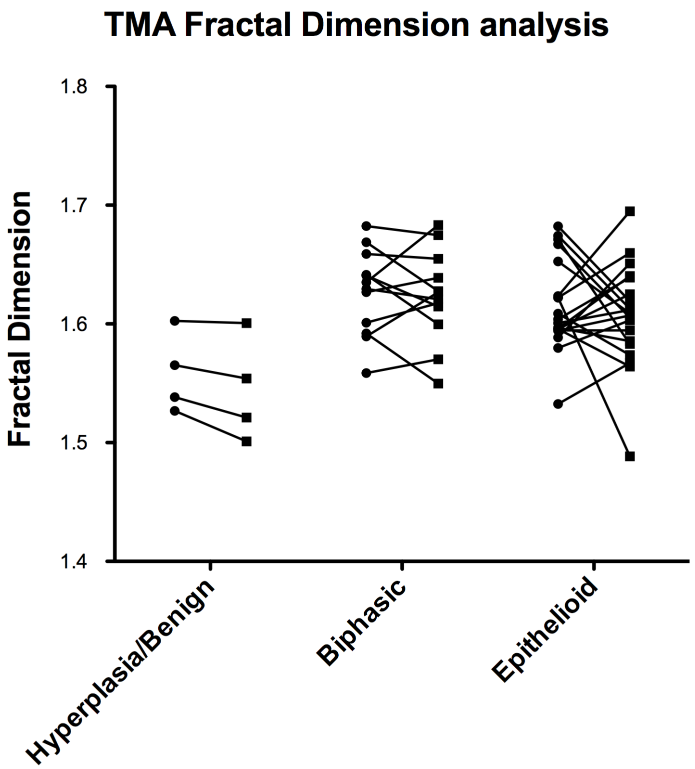

b

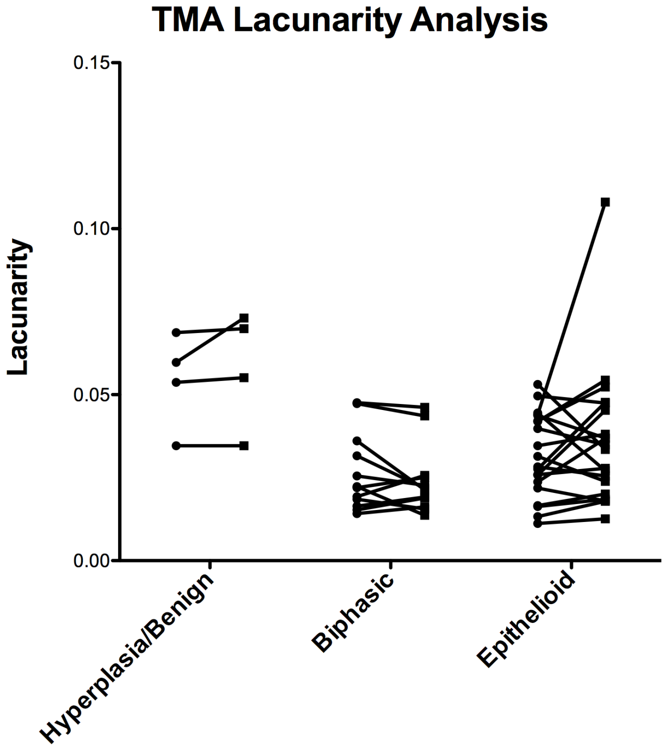

Supplemental Figure 2. ROC analysis of Fractal dimension classifier

a)

Biphasic Mesothelioma from benign by Fractal Dimension

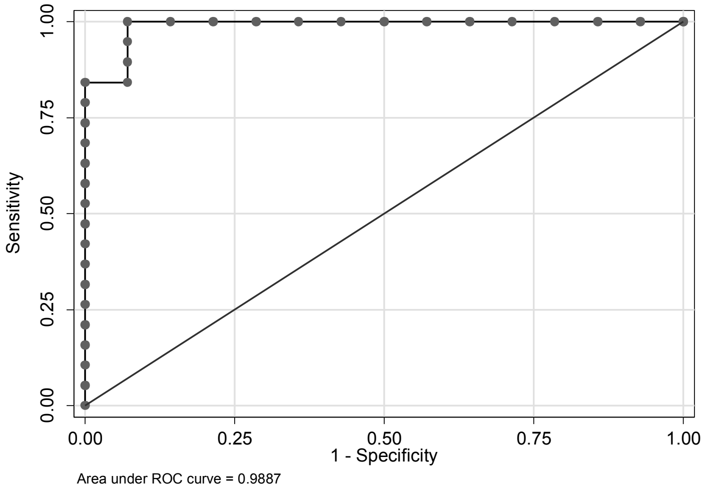

Cutoff	Sens	Spec	correctly classified
(>= 1.5644)	100.00%	92.86%	96.97%

b)

Epithelioid Mesothelioma from benign by Fractal Dimension

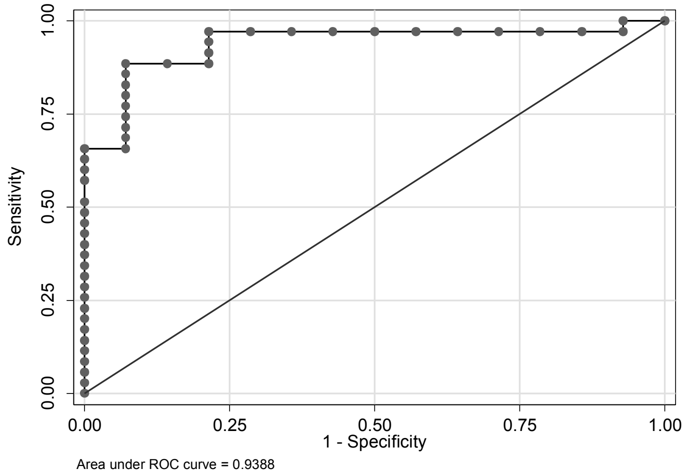

Cutoff	Sens	Spec	correctly classified
(>= 1.5426)	97.14%	78.57%	91.84%

Supplemental Figure 3. ROC analysis of Lacunarity classifier

a) Benign from Biphasic Mesothelioma by Lacunarity

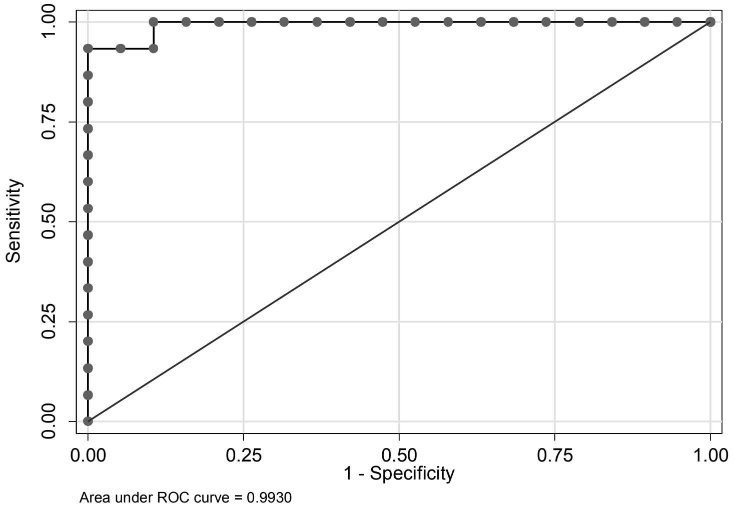

Cutoff	Sens	Spec	correctly classified
(>= .0518)	93.33%	100.00%	97.06%

b) Benign from Epithelioid Mesothelioma by Lacunarity

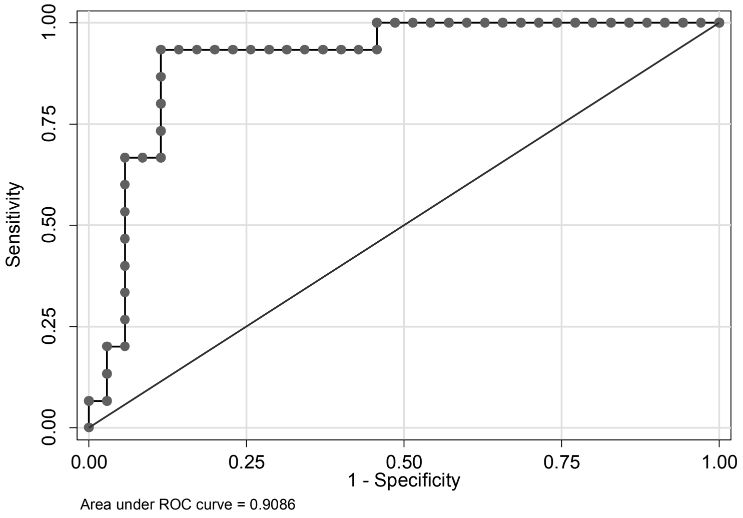

Cutoff	Sens	Spec	correctly classified
(>= .0518)	93.33%	88.57%	90.00%

Supplementary Data – Figure 4. Scatter plots of a)OCR and b)ECAR with inhibitor EC50 values

a) b)

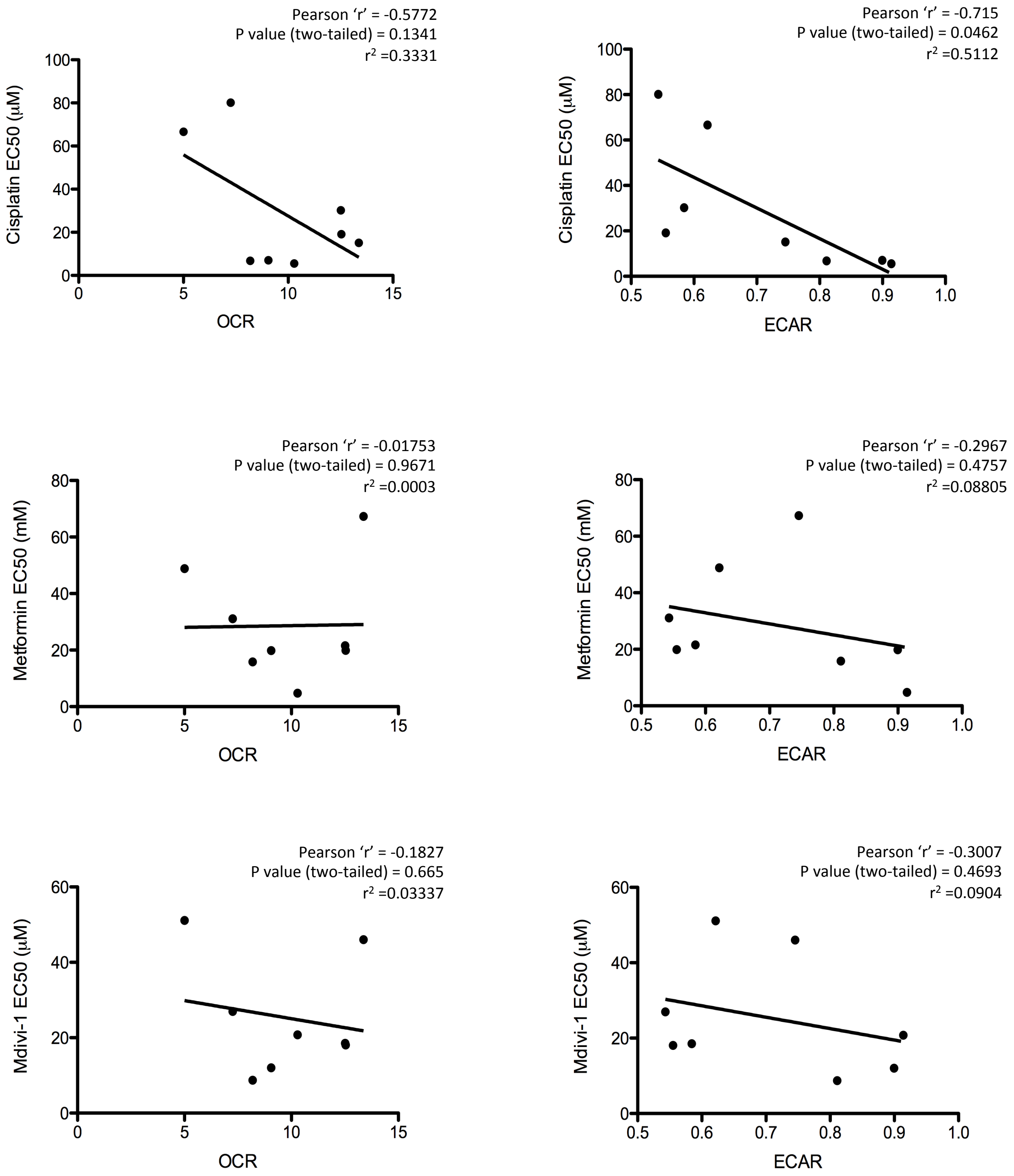

Supplementary Data – Figure 5. Expression of mitochondrial and metabolic regulatory proteins in malignant mesothelioma

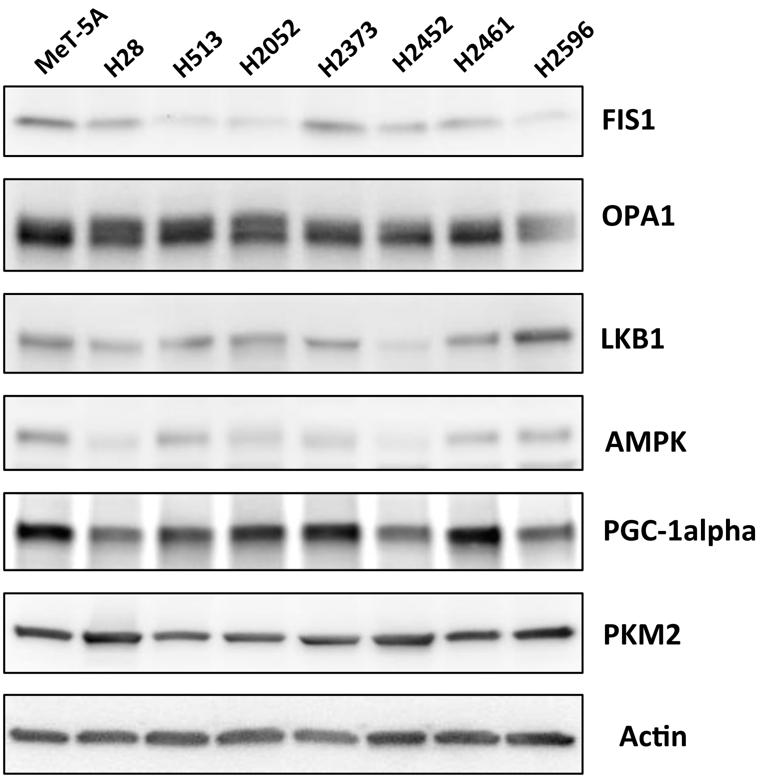

Supplement: Supplementary Information [file srep24578-s1.pdf]
